# The Mutator Phenotype: Adapting Microbial Evolution to Cancer Biology

**DOI:** 10.3389/fgene.2019.00713

**Published:** 2019-08-06

**Authors:** Federica Natali, Giulia Rancati

**Affiliations:** ^1^Institute of Medical Biology (IMB), Agency for Science, Technology and Research (A*STAR), Singapore, Singapore; ^2^School of Biological Sciences, Nanyang Technological University, Singapore, Singapore

**Keywords:** mutator phenotype, cell-to-cell heterogeneity, adaptation, selective pressure, asexually reproducing organisms

## Abstract

The mutator phenotype hypothesis was postulated almost 40 years ago to reconcile the observation that while cancer cells display widespread mutational burden, acquisition of mutations in non-transformed cells is a rare event. Moreover, it also suggested that cancer evolution could be fostered by increased genome instability. Given the evolutionary conservation throughout the tree of life and the genetic tractability of model organisms, yeast and bacterial species pioneered studies to dissect the functions of genes required for genome maintenance (caretaker genes) or for cell growth control (gatekeeper genes). In this review, we first provide an overview of what we learned from model organisms about the roles of these genes and the genome instability that arises as a consequence of their dysregulation. We then discuss our current understanding of how mutator phenotypes shape the evolution of bacteria and yeast species. We end by bringing clinical evidence that lessons learned from single-cell organisms can be applied to tumor evolution.

## Introduction

The mutator phenotype was proposed by Loeb almost 40 years ago to reconcile the observations that while cancer cells display widespread DNA and chromosomal changes (Alexandrov et al., 2013; Loeb, 2016), the rate of spontaneous mutation in somatic cells is low (Milholland et al., 2017). This hypothesis also suggested that an increased genome instability favors cancer evolution. Indeed, by reshuffling cancer cell genomes, the mutator phenotype generates cell-to-cell heterogeneity, which is the presence of cells with different genotypes and phenotypes within a population (Loeb, 2016). Since clonal competition within a tumor mass favors the expansion of fitter cells, the presence of cells with different phenotypes within a cancer sample increases the likelihood that some of them might be more aggressive and ultimately leads to poor patient survival (Greaves, 2015; McGranahan and Swanton, 2017). Thanks to the ease with which they can be grown in the laboratory and the wealth of genetic resources, in the last decades, model organisms have been extensively used to identify and dissect the function of genes required for genome stability. For instance, some of the available yeast genome-wide libraries include the systematic knockout collection (Winzeler et al., 1999; Giaever et al., 2002), the green fluorescent protein-tagged collection (Huh et al., 2003), and loss-of-function alleles for essential genes (Ben‐Aroya et al., 2008; Breslow et al., 2008; Li et al., 2011). Though progress in our ability to perform high-throughput screens and to manipulate mammalian cells has greatly improved (Behan et al., 2019), it is still difficult to envision a close future in which large-scale screens performed in model organisms could be easily and cost-effectively reproduced in mammalian cells. A more effective way is to directly translate findings of interesting candidates from model organisms to mammalian cells. For instance, a recent screen in budding yeast showed that, contrary to what was previously thought, heterozygous mutations in gatekeeper genes can cause genome instability (Coelho et al., 2019). Introduction of these mutations in human orthologs triggered genome instability also in human cells (Coelho et al., 2019). Interestingly, only some of the identified genes have been previously found mutated in cancer cells, supporting the idea that findings in budding yeast hold great potential for cancer cell biology.

## Cells Losing Balance

Studies on model organisms have been instrumental to understand mechanisms generating cell-to-cell heterogeneity and its consequence in evolutionary outcomes. While an important route to heterogeneity in yeast is sexual outbreeding (Long et al., 2015; Vazquez‐Garcia et al., 2017; Li et al., 2019), this point will not be discussed as evolution of cancer cells is comparable to evolution of asexually reproducing organisms. A route to heterogeneity common between single-cell model organisms and cancer cells involves the dysregulation of the genome integrity network. Initial identification of players of this network in yeast and bacteria was soon followed by the realization that cancer cells carry recurrent mutations in their respective human orthologs and provided ground for key findings. Some popular examples include the identification in cancer cells of cohesion mutations (Hill et al., 2016), of the links between microsatellite instability and the mismatch repair pathway (Kolodner and Marsischky, 1999), and of predicting novel therapeutic targets based on synthetic lethality (Ashworth et al., 2011; Beijersbergen et al., 2017). Though most of the core machinery required for genome maintenance and replication is conserved throughout evolution, there are important exceptions. The most notable differences include the lack of a well-defined nucleotide sequence of the origin of replication in human cells (Gerhardt et al., 2006) and the presence of the human DNA replication inhibitor, geminin (Symeonidou et al., 2012). Yeast cells also lack obvious orthologs of key human DNA repair enzymes, such as breast related cancer antigens 1/2 (BRCA1/2) or poly (ADP-ribose) polymerase (PARP) (Morales et al., 2014; Walsh, 2015). Given the therapeutic importance of some of these proteins, “humanized” yeast strains carrying cancer-associated mutations in BRCA genes have been generated (Guaragnella et al., 2014; Maresca et al., 2018) and used to screen for novel therapeutics.

Due to space constraints, we briefly summarize the role of key DNA repair genes and DNA replication genes. For a detailed overview of the field, please refer to the reviews by Kunkel and Erie (2015), Lujan et al. (2016), and Liu et al. (2017). For a summary of the genes discussed in this paragraph, please refer to **Table 1**.

**Table 1:** The table reports a list of mutations discussed in the section “Cells losing balance” and details phenotypic consequences arising from such mutations.

Gene	*S. cerevisiae* mutator allel	Type of mutation	Mutated domain	Affected function	Phenotype	Human mutator allele	Clinical relevance
*POL3*	pol3-D321G (Murphy et al., 2006)	Amino acid substitution (Murphy et al., 2006)	ExoI motif (Murphy et al., 2006)	Exonuclease proofreading activity (Murphy et al., 2006)	Increased forward mutation rate at *CAN1* gene (33-fold), reversion at trp1 locus (13-fold), and reversion at his2 locus (100-fold) compared to WT (Murphy et al., 2006)	POLD1-D316G (Barbari and Shcherbakova, 2017)	Mutation identified in colorectal cancer and endometrial cancer (Barbari and Shcherbakova, 2017)
	pol3-C324Y (Murphy et al., 2006)	As above (Murphy et al., 2006)	As above (Murphy et al., 2006)	As above (Murphy et al., 2006)	As above (Murphy et al., 2006)	POLD1-C319Y (Barbari and Shcherbakova, 2017)	Mutation observed in multiple myeloma and brain tumor (Barbari and Shcherbakova, 2017)
	pol3-L612M (Nick McElhinny et al., 2007)	As above (Nick McElhinny et al., 2007)	DNA polymerase motif (Nick McElhinny et al., 2007)	Partitioning of mismatches to the exonuclease active site (Nick McElhinny et al., 2007)	Increased forward mutation rate at *CAN1* gene (10-fold), compared to WT (Nick McElhinny et al., 2007)	L606M (Shlien et al., 2015)	Mutation observed in biallelic mismatch repair deficiency child brain tumor (Shlien et al., 2015)
	pol3-R696W (Daee et al., 2010)	As above (Daee et al., 2010)	DNA polymerase motif (Daee et al., 2010)	Fidelity of nucleotide incorporation (Daee et al., 2010)	Increased forward mutations at *CAN1* locus (65- to 200-fold) compared to WT (Daee et al., 2010)	POLD1-R689W (Mertz et al., 2017)	Mutation identified in the colon cancer cell line DLD1 (Mertz et al., 2017)
*MSH2*	msh2-G693S (Drotschmann et al., 1999)	As above (Drotschmann et al., 1999)	Walker A motif of *MSH2* (Drotschmann et al., 1999)	Recognition of base–base mispairs and indels of various size (Drotschmann et al., 1999)	Increase in reverse mutations at lys2::InsE-A14 locus (44- to 10,000-fold) compared to WT (Drotschmann et al., 1999)	hMSH2-G674S (Gammie et al., 2007)	Mutation associated with hereditary nonpolyposis colorectal cancer (HNPCC) (Gammie et al., 2007)
*MSH2–MSH6*	*MSH2–MSH6* co-overexpression (Chakraborty et al., 2018)	Overexpression (Chakraborty et al., 2018)		Efficiency of other DNA damage repair pathways due to sequestration of factors, such as PCNA (Chakraborty et al., 2018)	As above (Chakraborty et al., 2018)	hMSH2–hMSH6 copy number amplification (Wagner et al., 2016)	Overexpression of MSH2 and MAH6 in oral squamous cell carcinoma from patient’s biopsy correlates with poor prognosis (Wagner et al., 2016)
*MLH1*	mlh1-G64R (Clyne et al., 2009)	Amino acid substitution (Clyne et al., 2009)	ATP binding domain of *MLH1* (Clyne et al., 2009)	Exonuclease activity (Clyne et al., 2009)	Increase in forward -mutations at *CAN1* locus (4- to 8-fold) and reverse mutations at -lys2::InsE-A14 locus (4,000 to 8,000-fold) compared to WT (Clyne et al., 2009)	hMLH1-G67R (Clyne et al., 2009)	Mutation identified in patients with HNPCC (Clyne et al., 2009)
	mlh1-G64E (Clyne et al., 2009)	As above (Clyne et al., 2009)	As above (Clyne et al., 2009)	As above (Clyne et al., 2009)	As above (Clyne et al., 2009)	hMLH1-G67E (Clyne et al., 2009)	Mutation identified in a patient with a family history of atypical cancers, carrying male breast cancer, leiomyosarcoma of the thigh, colon cancer, and prostate cancer (Clyne et al., 2009)
*MLH1*	*MLH1* overexpression (Shcherbakova and Kunkel, 1999)	Overexpression from the natural promoter or from ADH1 promoter (Shcherbakova and Kunkel, 1999)		Formation of MMR complexes due to excessive binding with Mlh1 (Shcherbakova and Kunkel, 1999)	Increase in forward mutations at *CAN1* locus (2- to 26-fold), reverse mutations at lys2::InsE-A14 (100 to 8,500-fold) and his7-2 (5- to 170-fold) compared to WT (Shcherbakova and Kunkel, 1999)	Overexpression of *MLH1* is triggered by increased genomic damage (Wilczak et al., 2017)	Mlh1 overexpression correlated with genetic instability, advanced tumor stage, and poor outcome in patients with prostatic cancer (Wilczak et al., 2017)
	Δ mlh1 (Shcherbakova and Kunkel, 1999)	Homozygous deletion (haploid) (Shcherbakova and Kunkel, 1999)		Exonuclease activity (Shcherbakova and Kunkel, 1999)	As above (Shcherbakova and Kunkel, 1999)	Reduced expression of *MLH1* due to promoter hypermethylation (Gausachs et al., 2012)	Downregulation of MLH1 associated with the promoter hypermethylation observed in Lynch syndrome patients (Gausachs et al., 2012)
	MLH1/Δ mlh1 (12)	Heterozygous deletion (diploid) (Shcherbakova and Kunkel, 1999)		As above (Shcherbakova and Kunkel, 1999)	As above (Shcherbakova and Kunkel, 1999)	As above (Gausachs et al., 2012)	As above (Gausachs et al., 2012)

ADH1, alcohol dehydrogenase 1; PCNA, proliferating cell nuclear antigen; WT, wild type.

### Mutations Affecting DNA Polymerases

DNA replication of the lagging and the leading strands depends on the activity of two high-fidelity DNA polymerases, DNA polymerases δ (Polδ) and ε (Polε), respectively (Maslowska et al., 2018). The faithfulness of the process relies on the accuracy of nucleotide incorporation coupled with the 3′–5′ exonuclease proofreading activity (Pavlov and Shcherbakova, 2010). Indeed, biochemical assays showed that while purified human Polδ catalyzes one base substitution error every 22,000 nucleotides, the error rate decreased at least 10-fold in presence of a functional proofreading domain (Schmitt et al., 2009). Mouse models lacking a functional Polδ proofreading activity develop spontaneous cancers at high frequency (Goldsby et al., 2002), confirming *in vivo* the importance of the domain for genome stability and cancer formation. Moreover, germline mutations in the proofreading domain of Polδ and Polε have been identified in a number of families with increased susceptibility to colorectal adenomas and carcinomas (Palles et al., 2013; Heitzer and Tomlinson, 2014). Accordingly with an inability to repair mispaired bases inserted during DNA replication, tumors from affected patients increased the rate of base substitution mutations while maintaining microsatellite stability (Palles et al., 2013). Mutations in the proofreading activity of *S. cerevisiae* that mimic mutations in tumors resulted in a mutator phenotype and elevated spontaneous base substitution rates (Murphy et al., 2006; Nick McElhinny et al., 2007).

However, Polδ and Polε mutations outside of the proofreading domain were also mapped in sporadic cancers and cancer cell lines (Briggs and Tomlinson, 2013). Introduction of one of such variant, pol3-R696W (human POLD1-R689W), in heterozygosity in *S. cerevisiae* increased 30-fold the rate of forward mutations. At a biochemical level, pol3-R696W was shown to be an error-prone DNA polymerase with an increased nucleotide misinsertion rate and a specific mutational pattern (Daee et al., 2010) that is consistent with the one observed in colorectal cancer lines bearing the POLD1-R689W variant (Mertz et al., 2017). Collectively, these observations suggest that mutations affecting both the polymerase and the 3’–5’ exonuclease domains confer a mutator phenotype that can be translated from bacteria and yeast to human cells.

### Mutations Affecting Mismatch Repair Genes

The mismatch repair (MMR) pathway is a conserved surveillance system that recognizes and resolves misincorporated bases (Fishel, 2015). In prokaryotes, the MMR machinery is relatively simple and involves proteins detecting DNA mismatches (MutS), processing the damage (MutH), and bridging these two proteins together (MutL) (Fukui, 2010). While mutations in *mutS* and *mutL* human orthologs were found in the germline of patients with hereditary nonpolyposis colorectal cancer/Lynch syndrome (HNPCC/LS), and other cancer-predisposing Lynch variant syndromes (Lynch and de la Chapelle, 2003; Morales-Burgos et al., 2008; Wimmer and Kratz, 2010), they also somatically occur in up to 15% of sporadic colorectal, gastric, or endometrial carcinomas (Boland et al., 1998). Experimentally, engineered mice lacking functional MMR proteins are genomically unstable and predisposed to spontaneous cancer onset (Lee et al., 2016). Modeling cancer-related MMR mutations in yeast has been instrumental to dissect the consequences on cellular physiology of a non-functional mismatch repair pathway. For instance, mimicking MMR mutations found in HNPCC (Kurzawski et al., 2002) in yeast cells caused an increase in the rate of spontaneous (Drotschmann et al., 1999) and forward mutations (Clyne et al., 2009). Moreover, consistent with the observation that human cancer cell lines with dysregulation in the expression of the MMR proteins are genomically unstable (Ryan et al., 2017; Wilczak et al., 2017), tinkering with the expression levels of the yeast orthologs in *S. cerevisiae* resulted in significant increase of repeats’ instability and forward mutations (Shcherbakova and Kunkel, 1999; Chakraborty et al., 2018).

## The Rise and the Fall of the Mutator Phenotype

While maintenance of genome stability is key for reproductive success of prokaryotes and eukaryotes, laboratory and clinical evidence suggests that tinkering with such pathways favors cellular adaptation and population expansion during exposure to challenges. Below we discuss some such evidence.

### Lessons from Model Organisms

**Figure 1:**
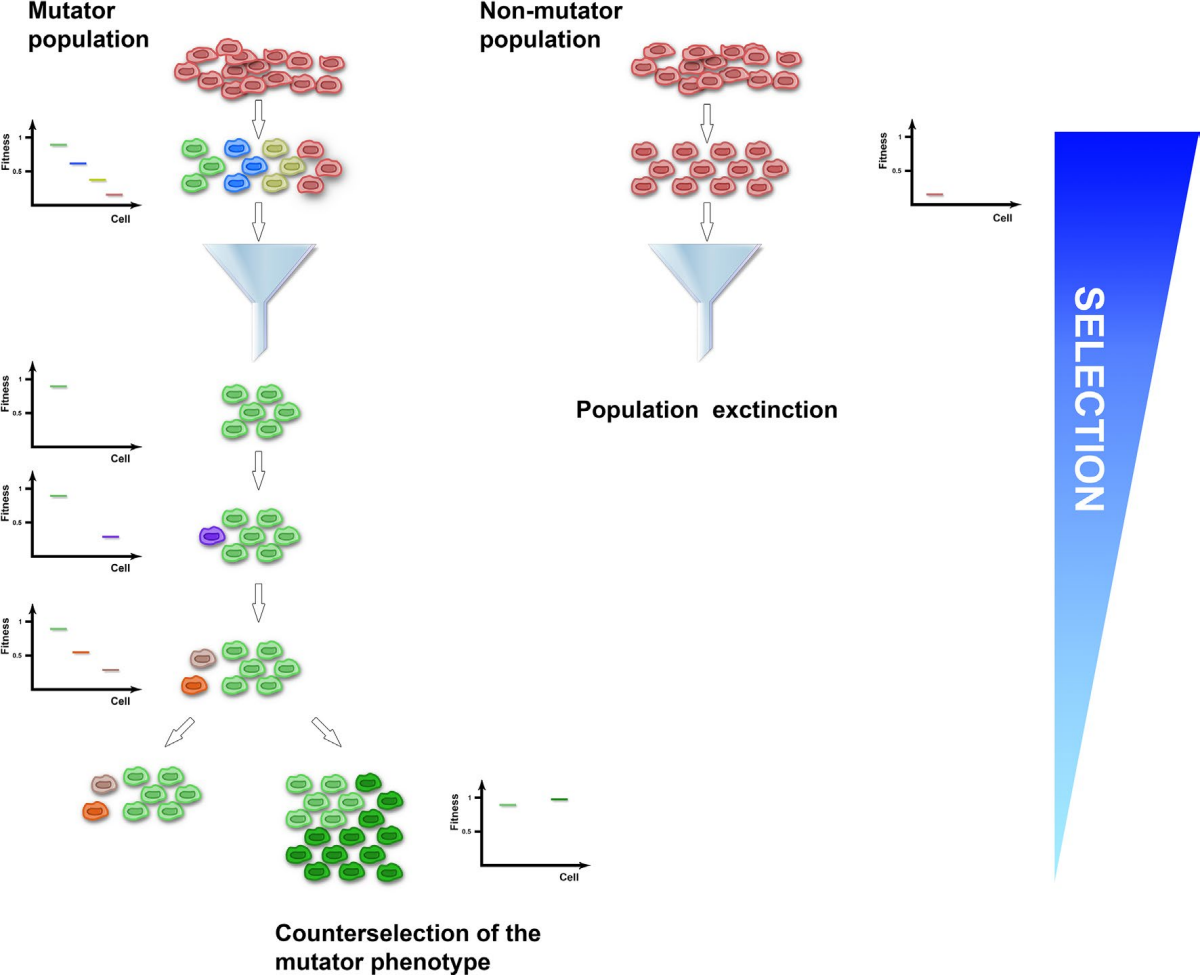
The mutator population (left) experiences enhanced genome instability and acquires cell-to-cell heterogeneity, while the non-mutator population (right) expands clonally. Upon application of selection, the mutator-induced phenotypic variation increases the probability of the population to have cells with a selective advantage (green cells) that could be fixed. Conversely, the clonal non-mutator population has higher probability of becoming extinct (red cells). Once adaptive mutations have been fixed and the population reaches a local optimum, acquisition of additional variation is detrimental and selected against (purple, orange, and grey cells). To increase adaptation in non-selective conditions, the mutator population can evolve a suppressor of the genome instability phenotype (dark green cells).

Several clinical isolates and natural populations of pathogenic bacteria and fungi were reported to have an enhanced mutation rate mostly mapped to defects in the methyl-directed mismatch repair system (Oliver et al., 2000; Bjorkholm et al., 2001; Chopra et al., 2003; Wang et al., 2013; Healey et al., 2016), suggesting that a mutator phenotype could be selected in fluctuating or hostile environments, such as the presence of drugs or adaptation to new ecological niches. Experimental studies have supported this idea. For instance, *MSH2*-defective *Cryptococcus neoformans*, *Candida glabrata*, and *Cryptococcus deuterogattii* strains increased mutation rates and underwent rapid adaptation to antifungal drugs (Healey et al., 2016; Billmyre et al., 2017; Boyce et al., 2017). Similarly, hypermutator *Staphylococcus aureus* bacteria strains impaired in the DNA mismatch repair pathway developed vancomycin resistance more rapidly than control strains (Schaaff et al., 2002). Moreover, mutant strains defective in DNA repair and characterized by increased mutation rates outcompeted wild-type strains and were fixed in 6 out of 12 *E. coli* populations in the Long-Term Evolution Experiment (Tenaillon et al., 2016). A link between increased mutation rates and adaptability comes also from observations that impairment in the activity of DNA repair pathways was often found to co-segregate with mutations conferring antibiotic resistance (Gould et al., 2007). At the theoretical level, a mutator phenotype potentially endows populations with a higher adaptability by generating cell-to-cell heterogeneity and a pool of allelic variants on which selection could select upon (**Figure 1**). Accordingly, mutator *msh2Δ S. cerevisiae* strains acquired resistance to the toxic arginine analog canavanine up to 20-fold faster than wild type (Bowers et al., 1999). Adaptive mutations encompassed single-nucleotide misincorporations and deletions of the canavanine influx pump gene (Sokolsky and Alani, 2000). Since such mutations are edited by the MMR pathway, these observations suggest that crippling with DNA replication or repair pathways could generate beneficial allelic variants. However, mutators with no direct effect on cellular fitness in asexually evolving unicellular organisms could sweep in a population if they are linked to beneficial mutations, a process called mutator hitchhiking. A large body of evidence coming from theoretical and experimental studies in both bacteria and yeast showed that the probability of hitchhiking has been linked to the population size and the fitness effects of beneficial mutations in complex-to-predict scenarios (Taddei et al., 1997; Notley-McRobb et al., 2002; Shaver et al., 2002; Wahl et al., 2002; Andre and Godelle, 2006; Thompson et al., 2006; Gerrish et al., 2007; Gentile et al., 2011; Raynes et al., 2014; Bui et al., 2015; Good and Desai, 2016). For instance, large population sizes are known to increase clonal interference, which has been shown to either delay or enhance fixation of mutators in different conditions (Raynes et al., 2014; Good and Desai, 2016). However, in populations at local fitness minima or experiencing fluctuating environments, mutators can hitchhike to a higher frequency when linked to strong beneficial mutations. It comes, therefore, as no surprise that mutator multidrug-resistant bacterial strains are a common feature of chronic infections, like cystic fibrosis or urinary tract infections (Oliver et al., 2000; Labat et al., 2005; Feliziani et al., 2010; Macia et al., 2014), where bacterial strains endure pulses of antibiotic treatments. In the laboratory, coupling of cycles of antibiotic or carbon source selection with mutagenesis increased the percentage of strains carrying mutations in mismatch repair genes up to 50–100% (Mao et al., 1997), further supporting the notion that fluctuating environments positively select for mutator strains. However, since the vast majority of mutations are detrimental, genome instability in populations at their fitness peaks comes with a cost (**Figure 1**). Indeed, a general role for asexual pathogenic mutators in the emergence of drug resistance is still being debated, possibly because mutators are selected against once beneficial mutations have been acquired. For instance, *mutS* mutant *S. aureus* laboratory strains characterized by a 78-fold increased mutation frequency did not increase the rate of adaptation to vancomycin (O’Neill and Chopra, 2002). Also, *MSH2* mutations in *C. glabrata* clades were shown to be present as polymorphisms within different natural populations that were equally sensitive or resistant to antifungal drugs (Carrete et al., 2018). At the same time, *C. glabrata* clinical isolates carrying *MSH2* mutations did not show increased resistance to azole or echinocandin (Singh et al., 2018). Lastly, an *in vivo* model for chronic bone infection in the rat showed that *MSH2* mutant *S. aureus* strains carried a decreased fitness and did not acquire antibiotic resistance (Daurel et al., 2007). The reported discrepancies on the effect of mutators on evolving populations of asexual singe-cell organisms could be linked to clonal interference, mutation effects, or population fitness or could be the result of the negative selection that mutators face once beneficial mutations have been acquired. Indeed, laboratory evidence showed that while mutator strains were initially selected for, both bacteria and yeast mutants experienced reduced transmission and recolonization abilities as well as rapid fitness decline upon prolonged passaging (Giraud et al., 2001; Trindade et al., 2010). These observations suggest that mutator strains could be counterselected once adaptation to the novel environment is achieved. Accordingly, mathematical modeling of *E. coli* population dynamics showed a sharp decline in the frequency of mutator strains once adaptation was achieved (Taddei et al., 1997). At a molecular level, experimental evolution correlated fitness drop of evolving strains with acquisition of detrimental mutations in genes required for optimal fitness (Andersson and Hughes, 1996; Funchain et al., 2000), suggesting that the mutational load of mutator strains could become a selective pressure itself. Accordingly, decreased cellular fitness after prolonged passaging of *msh2Δ* mutator *S. cerevisiae* strains in non-challenging environments was followed by restoration of genome stability by increasing the buffering ability of heat shock proteins (McDonald et al., 2012). Alternatively, restoration of genome stability arose either by acquisition of antimutator suppressor alleles or by replacing the mutator alleles with functional ones through horizontal gene transfer (Denamur et al., 2000; Wielgoss et al., 2013). Taken together, all of this evidence suggests that while the mutator phenotype is initially selected for during adaptation, it could be selected against once adaptive mutations are fixed.

### Evidence From Cancer Evolution

Intra- and inter-tumor heterogeneity has been observed in early (Jamal-Hanjani et al., 2017) as well as in advanced stages of tumor progression (Caswell and Swanton, 2017; Jamal-Hanjani et al., 2017). Its presence suggests that cancer proceeds through a branched evolutionary pathway (Nowell, 1976; McGranahan and Swanton, 2017). Specifically, single-cell–derived clones carrying different genomes, epigenomes, and karyotypes compete in a non-linear model that favors the expansion and the coexistence of clones containing distinct beneficial mutations under challenging environments (Merlo et al., 2006). The presence of cell-to-cell heterogeneity promotes cancer progression (Gerlinger et al., 2012; Loeb, 2016; McGranahan and Swanton, 2017) by potentially increasing the number of clones with penetrant driver mutations (Jamal-Hanjani et al., 2017), with resistance to drugs or poor environmental conditions (Scalerandi et al., 1999; Calcagno et al., 2010), or immune to interaction with host immune cells (Seliger, 2005).

Cancer cell populations evolve as asexually reproducing organisms and can be modeled as bacteria or mating-type locked laboratory yeast strains. As discussed above, studies on mutator populations in these model organisms indicate that while a mutator phenotype can initially promote adaptation to a variety of selective pressures, it has detrimental effects once adaptive mutations have been fixed. Does this dynamic also occur during cancer progression? We would like to propose this to be the case. In recent years, the tumultuous advances of deep-sequencing technologies increased our ability to perform and analyze large single-cell sequencing data sets (Gerlinger et al., 2012). By looking at mutations present in cancer cells in spatially distinct regions at different stages of cancer progression, a few lessons have emerged. First, consistent with a positive contribution of genome instability to cancer development and evolution in response to challenges, cancer cells display a high level of intra- and inter-tumor heterogeneity (McGranahan and Swanton, 2017). Experimentally, several mouse models of genome instability display an increased spontaneous incidence of cancer onset (Liu et al., 2007; Weaver et al., 2007) and increased tumor relapse when challenged by oncogene withdrawal (Sotillo et al., 2010). This suggests that mutator phenotype could increase aggressiveness or drug resistance of cancer cells. Second, early evolution stages of different types of cancers display genome instability. Thanks to the long latency and frequent biopsies patients are subjected to, one of the cancer types that undergoes the most frequent longitudinal sampling is Barrett’s esophagus. This neoplastic lesion frequently gives rise to esophageal adenocarcinoma and is associated with a high level of genomic instability (Reid et al., 2001). Consistent with the idea that mutator phenotype is an enabling characteristic of tumor development, heterogeneity of premalignant Barrett’s esophagus populations is a prognostic marker that correlates with increased probability of esophageal adenocarcinoma development (Maley et al., 2006). Another line of evidence that a mutator phenotype is an early event comes from clinical evidence that mutations in mismatch repair genes and genome instability occur early in HNPCC and colon cancer evolution. For instance, microsatellite instability was found in premalignant adenomas (Shibata et al., 1996), consistent with the idea that mutations in mismatch repair genes occur prior to hallmark mutation markers for colon cancer (Huang et al., 1996). Lastly, mathematical modeling favors a positive contribution of mutator phenotype in early events of cancer progression leading to rapid tumor growth (Beckman, 2009). Therefore, similarly to yeast and bacteria adaptation to hostile environments, the mutator phenotype can facilitate early stages as well as later stages of cancer evolution. Consistently, it was recently shown that metastatic cells have higher mutations rates than non-metastatic cancer cells (Bertucci et al., 2019). However, extreme genomic instability was reported to have a negative effect on tumor growth, leading to massive cancer cell death (Kops et al., 2004; Janssen et al., 2009). Similarly to what observed in model organisms, it was proposed that excessive mutational burden decreased cellular fitness as cells cannot tolerate high levels of genome instability (Komarova and Wodarz, 2004). Accordingly, clinical evidence suggests that high levels of chromosomal instability are a marker for better prognosis than intermediate ones in non-small-cell lung carcinoma (Jamal-Hanjani et al., 2015). Similar observations have been made in other epithelial tumors, such as ovarian and squamous non-small-cell lung cancer and gastric adenocarcinoma (Birkbak et al., 2011). Taken together, all of this clinical evidence suggests that cancer cells, pretty much like mutator yeasts, can evolve adaptive mechanisms to decrease the rate of genome instability once fitter and more aggressive cancer clones have emerged. This view is also supported by recent studies showing that at different stages of tumor progression, cancer cells exhibit distinct types of genome instability (Nik-Zainal et al., 2012). For instance, sequencing of spatially distant clear-cell renal carcinoma masses within patients showed that, while the bulk of the primary tumor was stable and diploid, cells from metastatic regions derived from a tetraploid intermediate and were genomically unstable (Gerlinger et al., 2012). Moreover, phylogenetic reconstruction of breast cancer tissues carrying BRCA mutations showed that while early mutations during cancer development were consistent with patients’ germline mutations, late-stage genome instability had a significantly different mutational pattern consistent with localized hypermutation with specific base substation (Nik-Zainal et al., 2012). Taken together, all of this clinical evidence suggests that different types of genome instability of tumor cells can be selected to better adapt to cycles of selective and non-selective environments as well as different selective pressures.

## Future Perspective

As speculated above, to better adapt to a variety of different selective and non-selective environments, cancer cells could tinker with their genome instability to either generate cell-to-cell heterogeneity or stabilize fitter clones or change their mutational landscape. Since different types of mutations could allow cells to differently hike the fitness landscape (Pavelka et al., 2010), the ability of cancer cells to switch between different mutational patterns could equip them with different “gears” to successfully adapt to challenges. Therefore, to successfully eradicate cancer cells, strategies to curb their incredible genome plasticity should be found. Given the similarity in the evolution of mutator phenotypes between single-cell model organisms and cancer cells, we predict that dissecting the molecular mechanisms that allow yeast or bacteria to fine-tune their genome instability will pinpoint targets to curb cancer genome plasticity.

## Author Contributions

FN and GR conceived and wrote the manuscript.

## Funding

This study is funded by the NRF Investigatorship (ref. no. NRF-NRFI05-2019-0008) awarded to GR.

## Conflict of Interest Statement

The authors declare that the research was conducted in the absence of any commercial or financial relationships that could be construed as a potential conflict of interest.
